# Eco-Friendly Superwetting Material for Highly Effective Separations of Oil/Water Mixtures and Oil-in-Water Emulsions

**DOI:** 10.1038/srep43053

**Published:** 2017-02-20

**Authors:** Chih-Feng Wang, Sheng-Yi Yang, Shiao-Wei Kuo

**Affiliations:** 1 Department of Materials Science and Engineering, I-Shou University, Kaohsiung, 840, Taiwan; 2 Department of Materials and Optoelectronic Science, National Sun Yat-Sen University, Kaohsiung, 804, Taiwan

## Abstract

Because the treatment of oily wastewater, generated from many industrial processes, has become an increasing environmental concern, the search continues for simple, inexpensive, eco-friendly, and readily scalable processes for fabricating novel materials capable of effective oil/water separation. In this study we prepared an eco-friendly superhydrophilic and underwater superoleophobic polyvinylpyrrolidone (PVP)-modified cotton that mediated extremely efficient separations of mixtures of oil/water and oil/corrosive solutions. This PVP-modified cotton exhibited excellent antifouling properties and could be used to separate oil/water mixtures continuously for up to 20 h. Moreover, the compressed PVP-modified cotton could separate both surfactant-free and -stabilized oil-in-water emulsions with fluxes of up to 23,500 L m^−2^ h^−1^ bar^−1^—a level one to two orders of magnitude higher than that possible when using traditional ultrafiltration membranes having similar rejection properties. The high performance of our PVP-modified cotton and its green, low-energy, cost-effective preparation suggest its great potential for practical applications.

Effective separations of oil/water mixtures and emulsions are challenges worldwide because of the expanding production of industrial oily wastewaters and the frequent oil spills that arise from industrial accidents and the sinking of oil tankers and other ships^1^,^2^,^3^,^4^,^5^. According to a report from the International Tanker Owners Pollution Federation (ITOPE), over 1800 large oil tanker accidents occurred from 1970 to 2015, resulting in the loss of approximately 5.72 million tons of oil. Traditional techniques for oil/water separations have low separation efficiencies and high energy costs, and are not applicable to the separation of oil/water emulsions^6^,^7^. As a result, there is a demand for more efficient methods for the separations of immiscible oil/water mixtures and emulsions. Recently, the use of superhydrophobic-superoleophilic (oil-removing) materials has become an effective and facile approach toward oil/water separations, through either selective filtration or absorption of the oil from the mixtures^8^,^9^,^10^,^11^,^12^,^13^,^14^,^15^,^16^. The first example of an oil/water separation system employing a superhydrophobic and superoleophilic material was reported in 2004 by Jiang and co-workers, based on a polytetrafluoroethylene (PTFE)-modified stainless-steel mesh^8^. Although such special wettable materials can be effective agents for oil/water separations, they are readily fouled, or even blocked up, by oils (especially high-viscosity oils) because of their intrinsic oleophilicity. The development of superhydrophilic and underwater-oleophobic (water-removing) materials may lead to a more practical, alternative, and feasible approach for oil/water separations.

Inspired by the wetting behavior of fish scales, Feng *et al*. described a superhydrophilic and underwater-superoleophobic hydrogel-coated mesh for the efficient separation of oil/water mixtures^17^. Following this strategy, several materials possessing both superhydrophilicity and underwater-superoleophobicity have been prepared, using various methods, with the aim of applying them in the efficient separations of oil/water mixtures and emulsions^18^,^19^,^20^,^21^,^22^,^23^,^24^,^25^,^26^,^27^,^28^,^29^. Feng and coworkers developed polydopamine and polyethylenepolyamine co-deposition films displaying superhydrophilicity and underwater-superoleophobicity for the effective separations of both immiscible oil/water mixtures and oil-in-water emulsions with high separation efficiencies^22^. Jin *et al*. used a salt-induced phase-inversion method to prepare superhydrophilic poly(acrylic acid)-g-poly(vinylidene fluoride) membranes that were applicable in the separations of both a surfactant-free emulsion and a surfactant-stabilized oil-in-water emulsion^23^. Underwater superoleophobic carbon nanotube-based materials have been prepared and applied in the separations of oil-in-water emulsions^25^. Nevertheless, multistep processes, stringent preparation specifications, and the use of harmful chemicals have limited the practical applications of all of these methods.

In this paper, we present a simple eco-friendly dipping method for the fabrication of a superhydrophilic and underwater-superoleophobic polyvinylpyrrolidone (PVP)-modified cotton. Cotton, a very common natural material, is one of the most favored fabrics for preparing clothes and textile products because it is soft, comfortable, breathable, and biodegradable and causes little skin irritation. PVP, which was first developed in Germany in 1930, has attracted an intense amount of interest from both academia and industry because of its high biological compatibility, nontoxicity, adhesiveness, facility to form complexes, and resistance to thermal degradation in solution. It has found widespread applicability in a number of areas—for example, in aerosol products (e.g., hair sprays), in pharmaceutical and cosmetic formulations, as a dispersing agent providing colloid stability, as a food additive, and also as a blood plasma substitute^30^,^31^,^32^,^33^. We found that PVP-modified cotton pre-wetted with water exhibits under-oil superhydrophilicity and water adsorption ability and could be used in effective oil/water separations. Our as-prepared PVP-modified cotton exhibited high separation capacity, allowing separation of oil/water mixtures continuously for up to 20 h without any increase in the oil content in the filtrate. Interestingly, the compressed PVP-modified cotton could separate both surfactant-free and -stabilized oil-in-water emulsions with high separation efficiencies. The excellent performance of this PVP-modified cotton in oil/water separations and its preparation through an eco-friendly process suggest that it has great potential for applicability in both academic and industrial settings.

## Results

**Figure 1:**
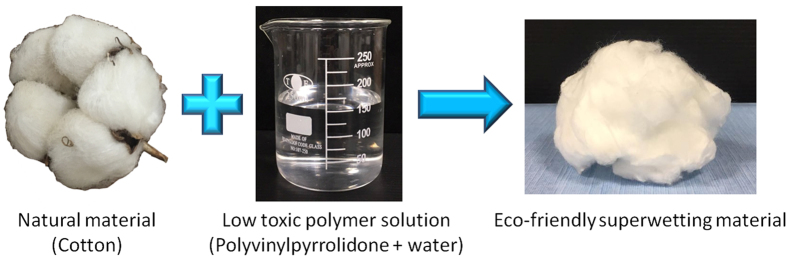
Preparation of the eco-friendly superwetting material.

**Figure 2:**
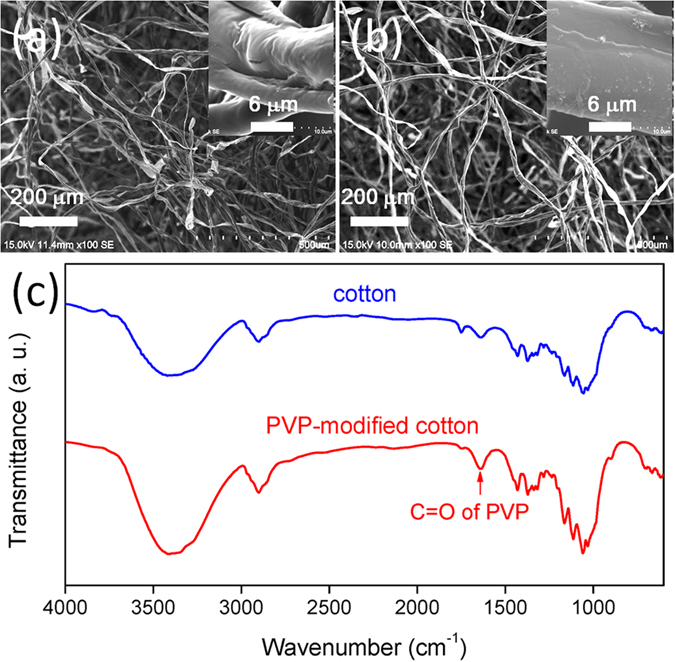
(**a**,**b**) SEM images of the (**a**) pristine and (**b**) PVP-modified cotton. (**c**) FTIR spectra of the pristine, PVP-modified cotton and PVP-modified cotton.

Cotton is an abundant bio-derived material that is environmentally friendly, of low toxicity, and of high chemical durability. In this study we prepared PVP-modified cotton through an eco-friendly one-step process without using any harmful chemicals (Fig. 1). We used SEM to study the morphologies of the cotton before (Fig. 2a) and after (Fig. 2b) modification with the PVP coating. The PVP-modified cotton had a morphology similar to that of the pristine material. Figure 2c presents ATR-FTIR spectra of the pristine and PVP-modified cotton samples. In contrast to the spectrum of the pristine cotton, a peak appeared at 1654 cm^−1^, assigned to the C=O groups of PVP, in the spectrum of the modified cotton. To investigate the wetting behavior of the PVP-modified cotton toward water and oil, we used a charge-coupled device camera system to record the spreading of water and oil droplets. When a water droplet (4 μL) came into contact with the surface, it spread out and permeated into the PVP-modified cotton instantly, resulting in a contact angle of approximately 0°. A similar situation occurred when using a droplet of oil as a detecting probe. Both processes were complete within 1 s, implying that the PVP-modified cotton possessed both superhydrophilicity and superoleophilicity (contact angles of *n*-hexane, *n*-hexadecane, isooctane, and diesel: all close to 0°) in air.

**Figure 3:**
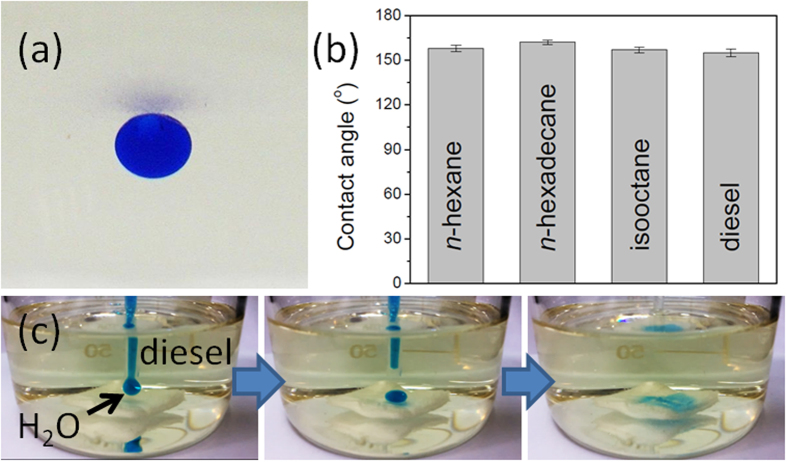
(**a**) Photograph of an oil droplet (*n*-hexadecane) beneath the PVP-modified cotton in water (contact angle: 162°). (**b**) Underwater contact angle of a series of oils. (**c**) Series of photographs displaying the spreading and permeating behavior of a water droplet on the pre-wetted PVP-modified cotton under oil.

Underwater oil droplets on the PVP-modified cotton surface were nearly spherical (Fig. 3a) and exhibited high contact angles (>150°; Fig. 3b)—typical underwater-superoleophobic properties. When we immersed the PVP-modified cotton in water, some water became trapped within its rough microstructure; subsequently, in the presence of oil, it formed an oil/water/solid composite interface. The trapped water molecules decreased the contact area between the oil and the PVP-modified cotton surface significantly, resulting in underwater-superoleophobicity. More importantly, the PVP-modified cotton exhibited excellent stability toward corrosive aqueous liquids. We tested the oleophobic stability of the PVP-modified cotton in 10 wt% NaCl, 1 M HCl, and 1 M NaOH. Underwater *n*-hexadecane droplets were nearly spherical on the PVP-modified cotton in 10 wt% NaCl, 1 M HCl, and 1 M NaOH, with oil contact angles of 160, 158, and 156°, respectively, revealing the stable underwater-superoleophobicity of the PVP-modified cotton in these corrosive liquids. The pre-wetted PVP-modified cotton (in the pre-wetting process, the PVP-modified cotton was immersed in water for 20 min and then squeezed to form a water layer on the skeleton of the PVP-modified cotton) exhibited superhydrophilicity, even under oil. Figure 3c reveals the high under-oil (diesel) wettability of a pre-wetted PVP-modified cotton: water droplets (4 μL) adsorbed immediately upon contacting the pre-wetted PVP-modified cotton. The under-oil superhydrophilicity and water adsorption ability of the pre-wetted PVP-modified cotton were also evident in various other organic solvents and oils (e.g., *n*-hexane, *n*-hexadecane, isooctane).

**Figure 4:**
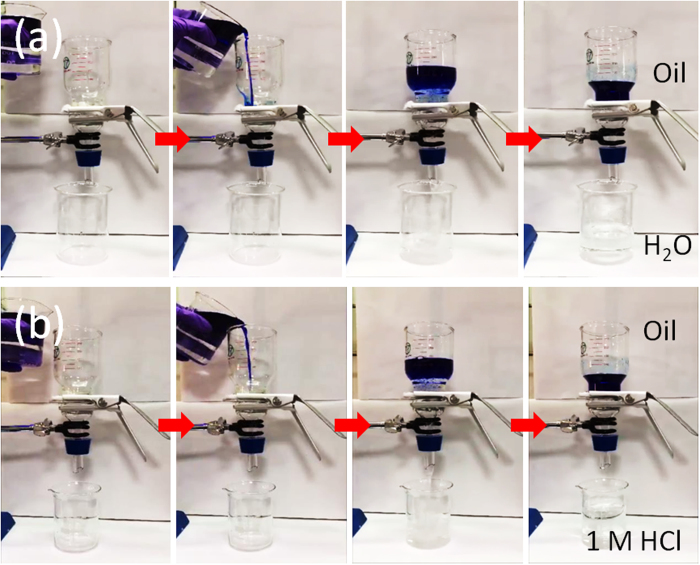
Solely gravity-driven separation of (**a**) oil/water and (**b**) oil/HCl_(aq)_ mixtures, performed through the PVP-modified cotton.

Because the PVP-modified cotton exhibited both underwater-superoleophobicity and water-permeability, it facilitated efficient separations of oil/water mixtures. Figure 4a displays the oil/water separation procedure. A 150 mL mixture of *n*-hexadecane and water (1:2, v/v) was poured onto the pre-wetted PVP-modified cotton (supported by a stainless-steel mesh), which was fixed between two vertical glass tubes. The water passed quickly through the pre-wetted PVP-modified cotton and entered the beaker below. Meanwhile, all of the oil was retained above the PVP-modified cotton, a result of the underwater-superoleophobicity of the PVP-modified cotton. The separation process was driven by gravity alone, with no other external force. Several other organic solvent/water mixtures, including those containing *n*-hexane, isooctane, and diesel, were also separated successfully through this approach (Figures S1,S2,S3). Next, we performed a continuous oil/water separation test to further study the robustness and antifouling properties of the PVP-modified cotton. In the continuous oil/water separation test (Figure S4), water was added into the upper glass tube continuously while maintaining the height of the oil/water mixture at 6.5 cm. Several oil/water mixtures, including those containing *n*-hexane, *n*-hexadecane, isooctane, and diesel, were separated through these continuous separation tests over a period of at least 1 h. We used a GC/FID system to measure the oil contents of the filtrates. The oil contents in the water samples separated from all of the immiscible oil/water mixtures were less than 7.0 ppm, indicating the high separation efficiency of the PVP-modified cotton. Table 1 reveals that each of the oil/water mixtures exhibited a high flux during the continuous separation test; the *n*-hexane/water, isooctane/water, *n*-hexadecane/water, and diesel/water mixtures provided comparable fluxes: 66,800, 61,200, 65,400, and 66,100 L m^−2^ h^−1^, respectively. Furthermore, continuous separations of *n*-hexadecane/water mixtures could be performed for up to 20 h through the PVP-modified cotton. No visible oil was observed in the collected water at any time during the testing process, confirming the high effectiveness of the separation of the oil/water mixture through the PVP-modified cotton. This result suggested that it should be possible to use the PVP-modified cotton to treat large amounts of oil/water mixtures over long periods of time, on account of the system’s excellent antifouling properties. Notably, most of the special wettable materials developed in previous studies have been used to separate oil/water mixtures comprising pure water and pure oil. Actual industrial production and waste emissions are often very complex—for example, strongly acidic, strongly basic, or containing a high concentration of salt. These complex systems pose a great challenge to oil/water separation systems. Accordingly, we prepared three kinds of oil/water mixtures—from *n*-hexadecane and 1 M HCl, 1 M NaOH, and 10 wt% NaCl, respectively—and separated them through the pre-wetted PVP-modified cotton (Figs 4b, S5 and S6). All of these aqueous solutions passed quickly through PVP-modified cotton but *n*-hexadecane was instead repelled and thereby held in the upper glass tube, demonstrating the robust underwater superoleophobicity of the PVP-modified cotton. The chemical inertness of our PVP-modified cotton suggests it might be useful for harsh environmental applications and could lead to important opportunities in industry and everyday life.

**Figure 5:**
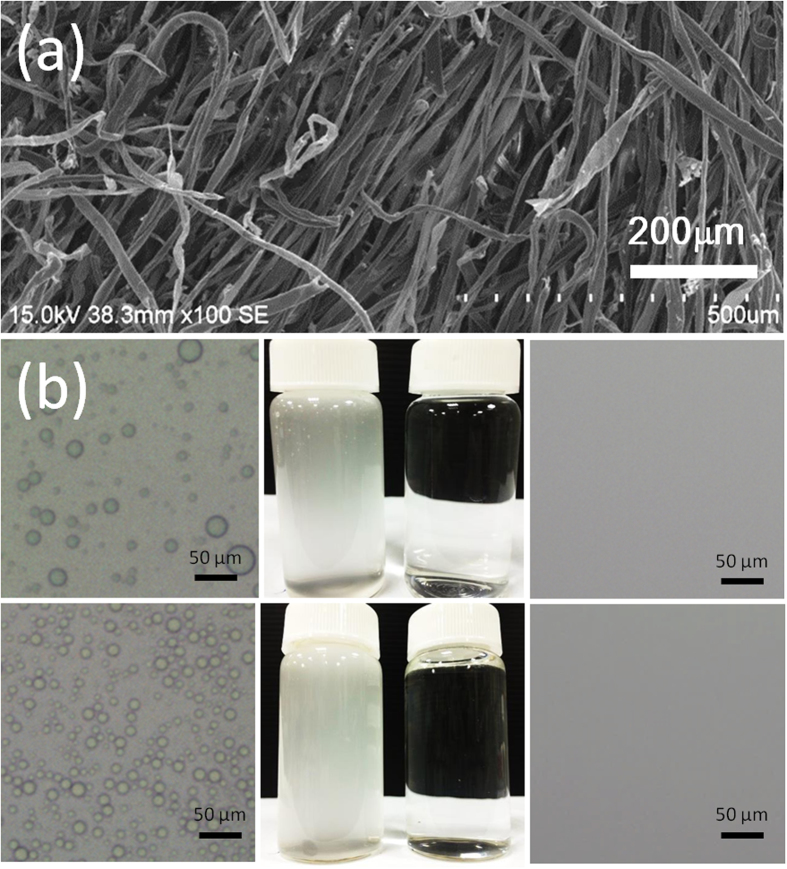
(**a**) SEM image of the compressed PVP-modified cotton. (**b**,**c**) Photographs of (**b**) surfactant-free and (**c**) -stabilized isooctane-in-water emulsions before and after separation using the compressed PVP-modified cotton.

**Figure 6:**
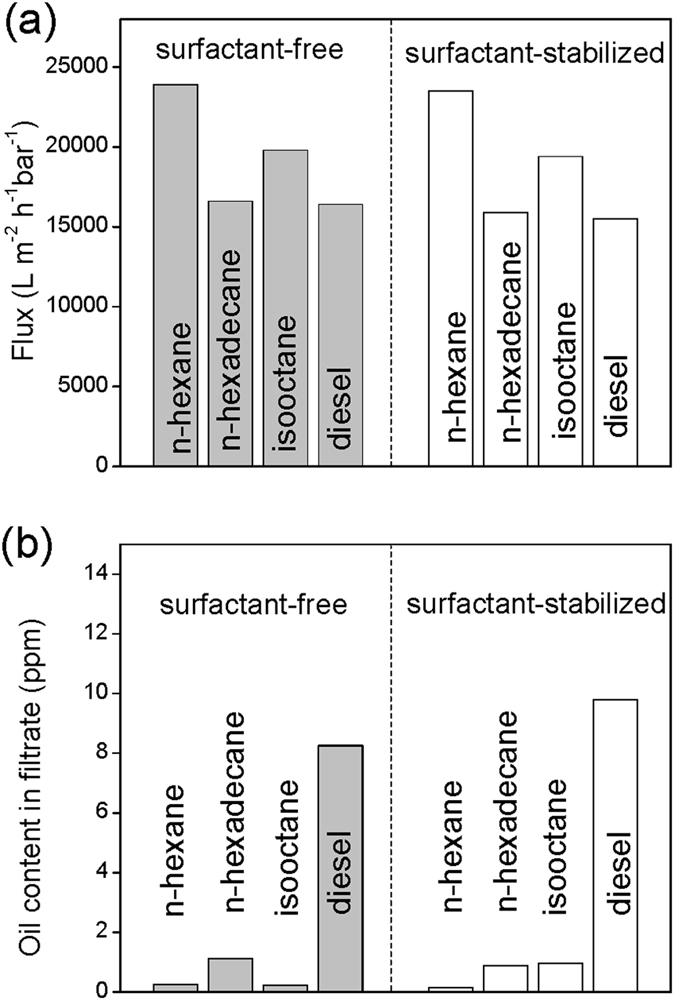
(**a**) Fluxes and (**b**) oil content in filtrates after separating various surfactant-free and -stabilized oil-in-water emulsions.

Wastewater containing emulsified oil/water mixtures is also a major environmental issue affecting a range of industries. Oil-in-water emulsions are more difficult to treat than oil/water mixtures, due to the microscale dimensions and good stability of emulsions, especially when stabilized by surfactants. Therefore, a critical need exists for novel materials that can separate emulsions with high efficiency. Interestingly, our PVP-modified cotton could be used for the successful separation of emulsified oil/water after applying a simple compression process. Figure 5a presents an SEM micrograph of the compressed PVP-modified cotton. Compared with the original film (Fig. 2b), the surface morphology changed such that the skeleton of the PVP-modified cotton was packed more compactly. Figure 5b reveals that the surfactant-free oil-in-water emulsion could be separated well and collected in a single step. We used optical microscopy to examine the separation effectiveness, determined by comparing the feed and its collected filtrate. The collected filtrate was transparent, unlike the feed, with no droplets appearing within it; in other words, the isooctane had been removed from the surfactant-free isooctane-in-water emulsion. For the oil-in-water emulsions including *n*-hexane, *n*-hexadecane, isooctane, and diesel, the fluxes were surprisingly high: 23,900, 16,600, 19,800, and 16,400 L m^−2^ h^−1^ bar^−1^, respectively (Fig. 6a). The compressed PVP-modified cotton also displayed high efficiency for the separation of surfactant-stabilized oil-in-water emulsions. The surfactant-stabilized oil-in-water emulsion was separated successfully in a single run (Fig. 5c). Similar to the results obtained for the separations of surfactant-free oil-in-water emulsions, again no oil droplets appeared in the image of the filtrate, confirming that the compressed PVP-modified cotton possessed high separation efficiency for the surfactant-stabilized oil-in-water emulsion. The compressed PVP-modified cotton exhibited extremely high separation fluxes for all of the various surfactant-stabilized oil-in-water emulsions incorporating *n*-hexane, *n*-hexadecane, isooctane, and diesel (23,500, 15,900, 19,400, and 15,500 L m^−2^ h^−1^ bar^−1^, respectively; Fig. 6a). These values are extremely high when compared with those of traditional ultrafiltration membranes with similar rejection properties. In addition to flux, high separation efficiency is another important evaluation index for a qualified emulsion separation material. As summarized in Fig. 6b, the oil contents in the collected filtrates from all of the separated emulsions were less than 7 ppm, confirming the system’s extremely high separation efficiency.

**Figure 7:**
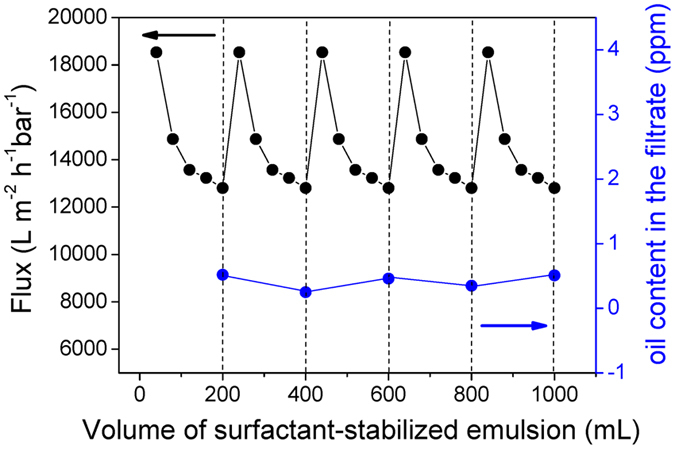
Real-time monitoring of the separation flux and oil purity in the filtrate during the cycles of a surfactant-stabilized isooctane-in-water emulsion separation test using the compressed PVP-modified cotton.

Oil fouling during oil/water separation is a common and tough issue for many filtration materials; it limits the reusability and decreases the separation efficiency. We evaluated the antifouling performance of our compressed PVP-modified cotton through a cyclic experiment of treatment of the surfactant-stabilized isooctane-in-water emulsion. For each cycle, the surfactant-stabilized emulsion (200 mL) was permeated through the compressed PVP-modified cotton and then the compressed PVP-modified cotton was simply washed with acetone. As revealed in Fig. 7, an obvious decrease in flux occurred upon increasing the emulsion volume permeating through the compressed PVP-modified cotton within one cycle; it was recovered completely, however, to the starting permeation flux after cleaning. The oil content was less than 1.0 ppm in each cycle. These results reveal the excellent recycling performance of the compressed PVP-modified cotton—almost no irreversible decline in water flux or increase in the oil content in the filtrated water occurred during the separation process. To assess the possible toxicity derived from the separation process through the PVP-modified cotton, we used ICP-MS to investigate the presence of metal elements in the original distilled water and corresponding collected filtrates. For all the elements considered, the detected values were all less than 0.01 ppm (Table 2). Thus, the PVP-modified cotton exhibited low toxicity and environmentally friendly properties.

## Discussion

We have fabricated an eco-friendly, low-toxicity superwetting material from a common bio-derived material (cotton) and a low-toxicity polymer (PVP). The PVP-modified cotton displayed superhydrophilicity and underwater-superoleophobicity; this material should have practical use in the effective separations of water-rich immiscible oil/water mixtures—even mixtures of oils and corrosive solutions—with extremely high separation efficiencies. The PVP-modified cotton exhibited excellent antifouling properties during long-term use. Moreover, the compressed PVP-modified cotton allowed effective separations of surfactant-free and -stabilized oil-in-water emulsions with ultrahigh fluxes (up to 23,900 L m^−2^ h^−1^ bar^−1^) and high separation efficiencies (oil contents in filtrated water: <10.0 ppm). Thus, this PVP-modified cotton has great potential for purifying immiscible oil/water mixtures and oil-in-water emulsions from industrial sources or found in our daily lives.

## Methods

### Materials

Tween 80 was supplied by Acros. PVP (*M*_w_ = 10,000) was supplied by Alfa Aesar.

### PVP modified cotton

A piece of degreasing cotton was soaked in 1.0 wt% aqueous PVP for 30 min. The treated sample was dried at 85 °C, cured at 150 °C for 5 min, and then washed multiple times with hot (50 °C) water. In the compression procedure, the PVP-modified cotton was compressed into a compact form having a density of 0.31 g cm^−3^.

### Oil-in-Water Emulsions

To prepare the surfactant-free oil-in-water emulsion, a mixture of oil (20 mL) and water (180 mL) was sonicated for 30 min. To prepare the surfactant-free *n*-hexadecane-in-water emulsions, the oil and water (1:15, v:v) mixtures were sonicated for 30 min to produce white solutions. To prepare the surfactant-stabilized oil-in-water emulsions, Tween 80 (0.02 g) was dissolved in water (200 mL), an oil (*n*-hexane, *n*-hexadecane, isooctane, or diesel; 2.0 mL) was added, and then the mixture was stirred for 1 h.

### Oil/Water Separation Experiment

A piece of PVP-modified cotton was supported by a stainless-steel mesh and then fixed between two glass vessels. All PVP-modified cotton samples used for oil/water separations were pre-wetted with water. The oil/water mixtures were poured into the filter and the separation was performed driven by gravity. The compressed PVP-modified cotton was fixed in a syringe. In the emulsion separation tests, a series of oil-in-water emulsions, including the surfactant-free and -stabilized emulsions, were passed through the compressed PVP-modified cotton under a suction vacuum pressure of 100 hPa.

### Instruments and Characterization

The microstructures of the pristine, PVP-modified, and compressed cottons were characterized using a HITACHI S-3400 scanning electron microscope (acceleration voltage: 15.0 kV). Attenuated total reflection Fourier transform infrared (ATR-FTIR) spectra of the pristine and PVP-modified cotton samples were recorded using a PerkinElmer Spectrum 100 FTIR spectrometer. Static contact angles of droplets (5 μL) were measured using an FDSA MagicDroplet-100 contact angle goniometer; each reported contact angle represents the average of six measurements. The oil contents in the filtrate water were determined using gas chromatography with flame ionization detection [GC/FID; 5890 (HP) and 7890 (Agilent)]. Optical microscopy images were recorded using an Olympus BX51M instrument after placing a drop of an emulsion solution onto a biological counting board. The presence of metal elements in the original water sample and corresponding collected filtrates was measured using inductively coupled plasma mass spectrometry (ICP-MS; PerkinElmer Optima 2100 DV).

## Additional Information

**How to cite this article**: Wang, C.-F. *et al*. Eco-Friendly Superwetting Material for Highly Effective Separations of Oil/Water Mixtures and Oil-in-Water Emulsions. *Sci. Rep.* **7**, 43053; doi: 10.1038/srep43053 (2017).

**Publisher's note:** Springer Nature remains neutral with regard to jurisdictional claims in published maps and institutional affiliations.

## Supplementary Material

Supplementary Video

Supporting Information

## Figures and Tables

**Table 1:** The oil content in filtrate and flux of the PVP-modified cotton during continuous oil/water separation.

Mixture	Oil content in filtrate (ppm)	Flux (L m^−2^ h^−1^)
*n*-hexane/water	6.70	66800
isooctane/water	0.70	61200
*n*-hexadecane/water	0.38	65400
diesel/water	3.06	66100

**Table 2:** ICP-MS trace element analyses of original distilled water and corresponding collected filtrates.

Element	Original distill water	Corresponding collected filtrates	Detection Limit (ppm)
Arsenic	ND	ND	0.01
Cadmium	ND	ND	0.01
Chromium	ND	ND	0.01
Copper	ND	ND	0.01
Lead	ND	ND	0.01
Nickel	ND	ND	0.01

ND = Not detected.

## References

[b1] Yip T. L., Talley W. K. & Jin D. The effectiveness of double hulls in reducing vessel-accident oil spillage. Mar. Pollut. Bull. 62, 2427–2432 (2011). 21924747 10.1016/j.marpolbul.2011.08.026

[b2] Dalton T. & Jin D. Extent and frequency of vessel oil spills in US marine protected areas. Mar. Pollut. Bull. 60, 1939–1945 (2010). 20797735 10.1016/j.marpolbul.2010.07.036

[b3] Li Q. X., Kang C. B. & Zhang C. K. Waste water produced from an oilfield and continuous treatment with an oil-degrading bacterium. Process Biochem. 40, 873–877 (2005).

[b4] Wu L., Ge G. & Wan J. B. Biodegradation of oil wastewater by free and immobilized Yarrowia lipolytica W29. J. Environ. Sci. 21, 237–242 (2009). 10.1016/s1001-0742(08)62257-3 19402428

[b5] Ahmad A. L., Sumathi S. & Hameed B. H. Coagulation of residue oil and suspended solid in palm oil mill effluent by chitosan, alum and PAC. Chem. Eng. J. 118, 99–105 (2006).

[b6] Huang X. F. & Lim T. T. Performance and mechanism of a hydrophobic-oleophilic kapok filter for oil/water separation. Desalination 190, 295–307 (2006).

[b7] Sayari A., Hamoudi S. & Yang Y. Applications of pore-expanded mesoporous silica. 1. Removal of heavy metal cations and organic pollutants from wastewater. Chem. Mater. 17, 212–216 (2005).

[b8] Feng L. . A super-hydrophobic and super-oleophilic coating mesh film for the separation of oil and water. Angew. Chem. Int. Ed. 43, 2012–2014 (2004). 10.1002/anie.200353381 15065288

[b9] Wang C. F. & Lin S. J. Robust Superhydrophobic/Superoleophilic Sponge for Effective Continuous Absorption and Expulsion of Oil Pollutants from Water. ACS Appl. Mater. Interfaces 5, 8861–8864 (2013). 24032484 10.1021/am403266v

[b10] Wang C. F., Tzeng F. S., Chen H. G. & Chang C. J. Ultraviolet-Durable Superhydrophobic Zinc Oxide-Coated Mesh Films for Surface and Underwater-Oil Capture and Transportation. Langmuir 28, 10015–10019 (2012). 22679902 10.1021/la301839a

[b11] Zhang W. F., Lu X., Xin Z. & Zhou C. L. A self-cleaning polybenzoxazine/TiO_2_ surface with superhydrophobicity and superoleophilicity for oil/water separation. Nanoscale 7, 19476–19483 (2015). 26530425 10.1039/c5nr06425b

[b12] Karatum O., Steiner S. A., Griffin J. S., Shi W. B. & Plata D. L. Flexible, Mechanically Durable Aerogel Composites for Oil Capture and Recovery. ACS Appl. Mater. Interfaces 8, 215–224 (2016). 26701744 10.1021/acsami.5b08439

[b13] Yang Y., Liu Z. J., Huang J. & Wang C. Y. Multifunctional, robust sponges by a simple adsorption-combustion method. J. Mater. Chem. A 3, 5875–5881 (2015).

[b14] Zhang W. F. . A Solvothermal Route Decorated on Different Substrates: Controllable Separation of an Oil/Water Mixture to a Stabilized Nanoscale Emulsion. Adv. Mater. 27, 7349–7355 (2015). 26489016 10.1002/adma.201502695

[b15] Gu J. C. . Robust preparation of superhydrophobic polymer/carbon nanotube hybrid membranes for highly effective removal of oils and separation of water-in-oil emulsions. J. Mater. Chem. A 2, 15268–15272 (2014).

[b16] Gu J. C. . Janus Polymer/Carbon Nanotube Hybrid Membranes for Oil/Water Separation. ACS Appl. Mater. Interfaces 6, 16204–16209 (2014). 25157932 10.1021/am504326m

[b17] Xue Z. X. . A Novel Superhydrophilic and Underwater Superoleophobic Hydrogel-Coated Mesh for Oil/Water Separation. Adv. Mater. 23, 4270–4273 (2011). 22039595 10.1002/adma.201102616

[b18] Chen Y. E. . A Co_3_O_4_ nano-needle mesh for highly efficient, high-flux emulsion separation. J. Mater. Chem. A 4, 12014–12019 (2016).

[b19] Zhu Y. Z. . A novel zwitterionic polyelectrolyte grafted PVDF membrane for thoroughly separating oil from water with ultrahigh efficiency. J. Mater. Chem. A 1, 5758–5765 (2013).

[b20] Chaudhary J. P., Vadodariya N., Nataraj S. K. & Meena R. Chitosan-Based Aerogel Membrane for Robust Oil-in-Water Emulsion Separation. ACS Appl. Mater. Interfaces 7, 24957–24962 (2015). 26485061 10.1021/acsami.5b08705

[b21] Chen P. C. & Xu Z. K. Mineral-Coated Polymer Membranes with Superhydrophilicity and Underwater Superoleophobicity for Effective Oil/Water Separation. Sci. Rep. 3, 2776 (2013). 24072204 10.1038/srep02776 PMC3784956

[b22] Cao Y. Z., Liu N., Zhang W. F., Feng L. & Wei Y. One-Step Coating toward Multifunctional Applications: Oil/Water Mixtures and Emulsions Separation and Contaminants Adsorption. ACS Appl. Mater. Interfaces 8, 3333–3339 (2016). 26751288 10.1021/acsami.5b11226

[b23] Zhang W. B. . Salt-Induced Fabrication of Superhydrophilic and Underwater Superoleophobic PAA-g-PVDF Membranes for Effective Separation of Oil-in-Water Emulsions. Angew. Chem. Int. Ed. 53, 856–860 (2014). 10.1002/anie.201308183 24307602

[b24] Yang H. C. . Silica-Decorated Polypropylene Microfiltration Membranes with a Mussel-Inspired Intermediate Layer for Oil-in-Water Emulsion Separation. ACS Appl. Mater. Interfaces 6, 12566–12572 (2014). 24998407 10.1021/am502490j

[b25] Gao S. J., Zhu Y. Z., Zhang F. & Jin J. Superwetting polymer-decorated SWCNT composite ultrathin films for ultrafast separation of oil-in-water nanoemulsions. J. Mater. Chem. A 3, 2895–2902 (2015).

[b26] Wang C. F., Huang H. C. & Chen L. T. Protonated Melamine Sponge for Effective Oil/Water Separation. Sci. Rep. 5, 14294 (2015). 26399444 10.1038/srep14294 PMC4585846

[b27] Fan J. B. . Directly Coating Hydrogel on Filter Paper for Effective Oil-Water Separation in Highly Acidic, Alkaline, and Salty Environment. Adv. Funct. Mater. 25, 5368–5375 (2015).

[b28] Zhou K. . Ultrathin cellulose nanosheet membranes for superfast separation of oil-in-water nanoemulsions. Nanoscale 6, 10363–10369 (2014). 25073443 10.1039/c4nr03227f

[b29] Zhang L. . Underwater superoleophobic carbon nanotubes/core-shell polystyrene@Au nanoparticles composite membrane for flow-through catalytic decomposition and oil/water separation. J. Mater. Chem. A 4, 10810–10815 (2016).

[b30] Immich A. P. S., de Araujo P. H. H., Catalani L. H., de Souza A. A. U. & Souza S. Crosslinking of Poly(N-vinyl-2-pyrrolidone) in the Coating of Cotton Yarn. Polym. Eng. Sci. 51, 445–453 (2011).

[b31] Fahmy H. M., Abo-Shosha M. H. & Ibrahim N. A. Finishing of cotton fabrics with poly (N-vinyl-2-pyrrolidone) to improve their performance and antibacterial properties. Carbohyd. Polym. 77, 845–850 (2009).

[b32] Hayama M., Yamamoto K., Kohori F. & Sakai K. How polysulfone dialysis membranes containing polyvinylpyrrolidone achieve excellent biocompatibility? J. Membrane Sci. 234, 41–49 (2004).

[b33] Robinson S. & Williams P. A. Inhibition of protein adsorption onto silica by polyvinylpyrrolidone. Langmuir 18, 8743–8748 (2002).

