# Supplementary material for: Eco-Friendly Superwetting Material for Highly Effective Separations of Oil/Water Mixtures and Oil-in-Water Emulsions
Source: Sci Rep. 2017 Feb 20;7:43053. doi: 10.1038/srep43053 (PMC5316977; doi:10.1038/srep43053)
Supplement: Supporting Information [file srep43053-s2.pdf]

Supporting Information

Eco-Friendly Superwetting Material for Highly Effective Separations of Oil/Water Mixtures and Oil-in-Water Emulsions

Chih-Feng Wang[a]*, Sheng-Yi Yang[a], Shiao-Wei Kuo[b]

a Department of Materials Science and Engineering, I-Shou University, Kaohsiung, 840, Taiwan.

b Department of Materials and Optoelectronic Science, National Sun Yat-Sen University, Kaohsiung, 804, Taiwan

*To whom all correspondence should be addressed

E-mail: cfwang@isu.edu.tw

Tel: 886-7-6577711-3129

Fax: 886-7-6578444

Supporting Information: Solely gravity-driven separation for *n*-hexane/water, isooctane/water, diesel/water, *n*-hexadecane/$NaOH_{(aq)}$, and *n*-hexadecane/$NaCl_{(aq)}$ mixtures, performed using the PVP-modified cotton. Video legend: video clips of the oil/water separation process through the PVP-modified cotton.

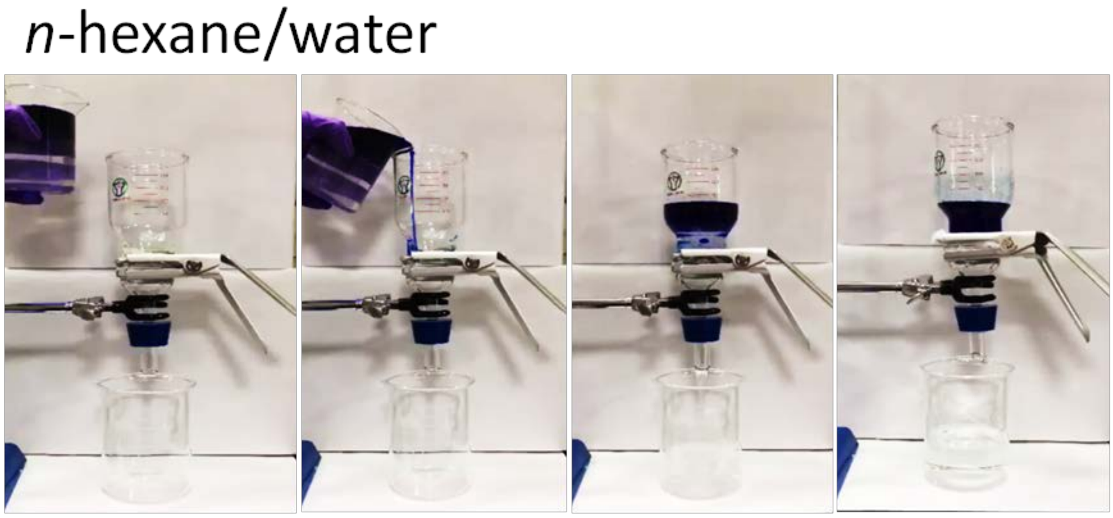

Figure S1. Solely gravity-driven separation of *n*-hexane/water mixture performed through the PVP-modified cotton.

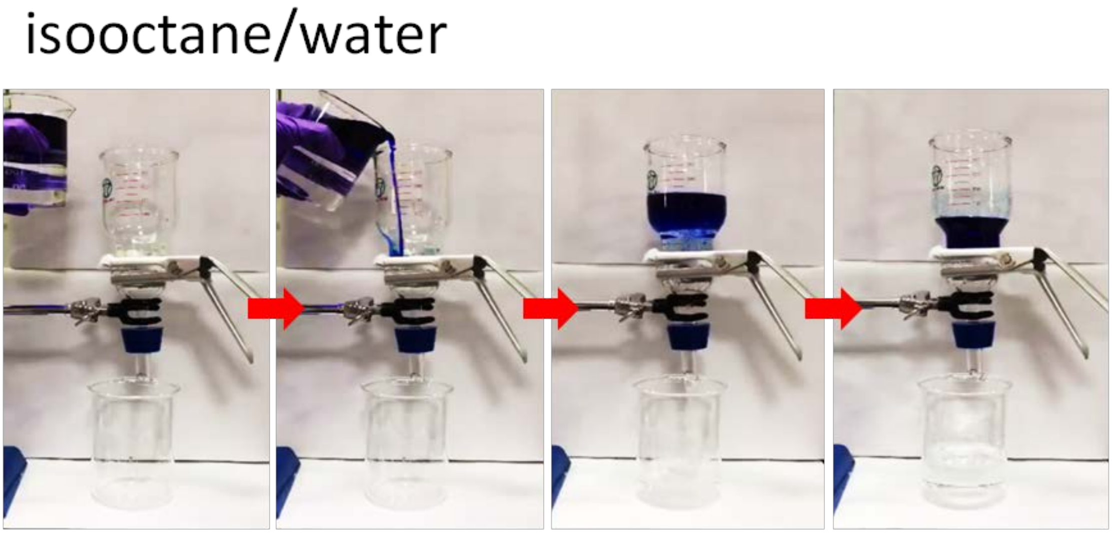

Figure S2. Solely gravity-driven separation of isooctane/water mixture performed through the PVP-modified cotton.

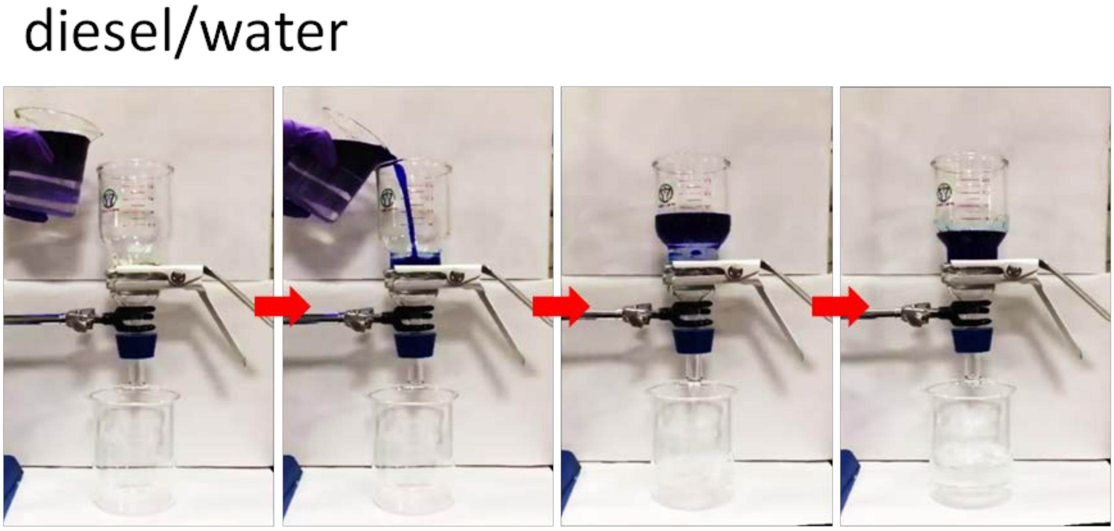

Figure S3. Solely gravity-driven separation of diesel/water mixture performed through the PVP-modified cotton.

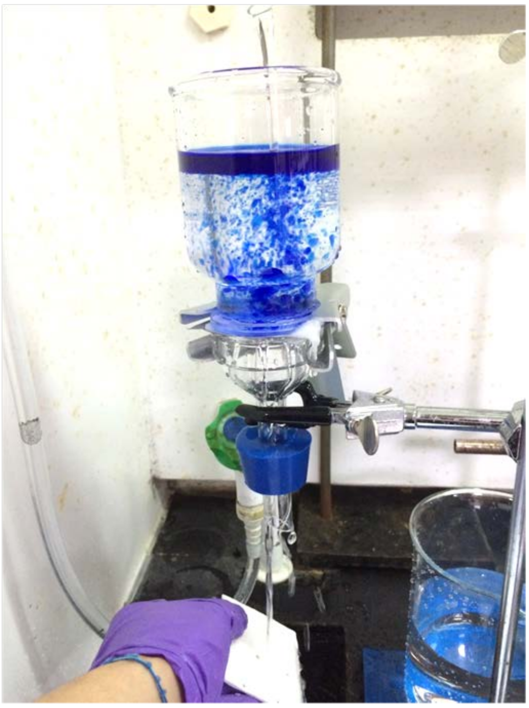

Figure S4. Continuous separation test for oil/water mixtures, performed using the PVP-modified cotton.

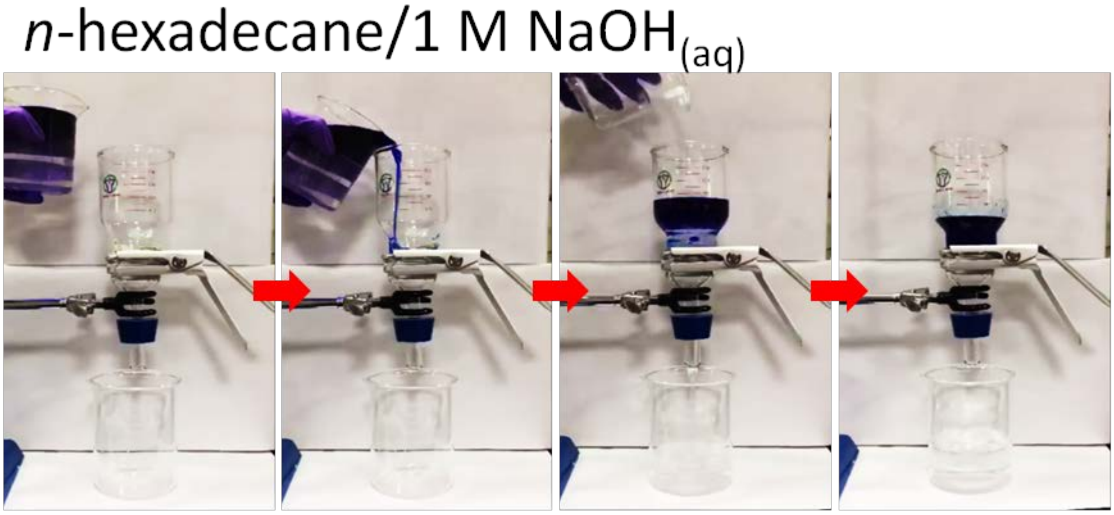

Figure S5. Solely gravity-driven separation of n-hexadecane/1 M $NaOH_{(aq)}$ mixture performed through the PVP-modified cotton.

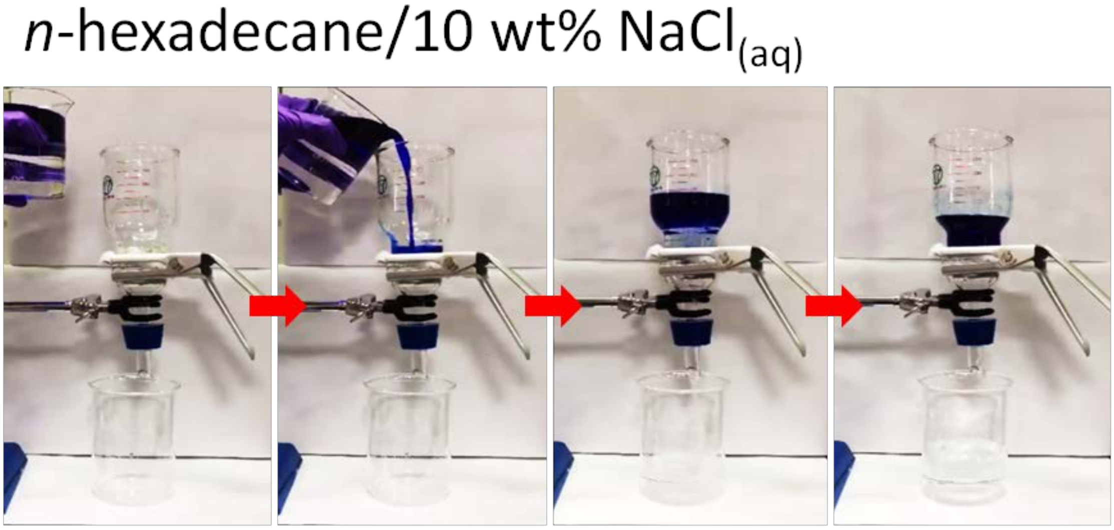

Figure S6. Solely gravity-driven separation of n-hexadecane/10 wt % $NaCl_{(aq)}$ mixture performed through the PVP-modified cotton.
